# Improving the quality of nursing documentation at a residential care home: a clinical audit

**DOI:** 10.1186/s12912-021-00629-9

**Published:** 2021-06-21

**Authors:** Preben Søvik Moldskred, Anne Kristin Snibsøer, Birgitte Espehaug

**Affiliations:** 1 Luranetunet Care Centre, Solstrandvegen 39, 5200 Os, Norway; 2 Centre for Evidence-Based Practice, Faculty of Health and Social Sciences, Western Norway University of Applied Sciences, Postbox 7030, 5020 Bergen, Norway; 3 Department of Health and Caring Sciences, Faculty of Health and Social Sciences, Western Norway University of Applied Sciences, Postbox 7030, 5020 Bergen, Norway

**Keywords:** Nursing, audit, electronic health records, nursing records

## Abstract

**Background:**

Quality in nursing documentation holds promise to increase patient safety and quality of care. While high-quality nursing documentation implies a comprehensive documentation of the nursing process, nursing records do not always adhere to these documentation criteria. The aim of this quality improvement project was to assess the quality of electronic nursing records in a residential care home using a standardized audit tool and, if necessary, implement a tailored strategy to improve documentation practice.

**Methods:**

A criteria-based clinical audit was performed in a residential care home in Norway. Quantitative criteria in the N-Catch II audit instrument was used to give an assessment of electronic nursing records on the following: nursing assessment on admission, nursing diagnoses, aims for nursing care, nursing interventions, and evaluation/progress reports. Each criterium was scored on a 0–3 point scale, with standard (complete documentation) coinciding with the highest score. A retrospective audit was conducted on 38 patient records from January to March 2018, followed by the development and execution of an implementation strategy tailored to local barriers. A re-audit was performed on 38 patient records from March to June 2019.

**Results:**

None of the investigated patient records at audit fulfilled standards for recommended nursing documentation practice. Mean scores at audit varied from 0.4 (95 % confidence interval 0.3–0.6) for “aims for nursing care” to 1.1 (0.9–1.3) for “nursing diagnoses”. After implementation of a tailored multifaceted intervention strategy, an improvement (*p* < 0.001) was noted for all criteria except for “evaluation/progress reports” (*p* = 0.6). The improvement did not lead to standards being met at re-audit, where mean scores varied from 0.9 (0.8–1.1) for “evaluation/progress reports” to 1.9 (1.5–2.2) for “nursing assessment on admission”.

**Conclusions:**

A criteria-based clinical audit with multifaceted tailored interventions that addresses determinants of practice may improve the quality of nursing documentation, but further cycles of the clinical audit process are needed before standards are met and focus can be shifted to sustainment of knowledge use.

## Background

Electronic nursing records have been argued as a tool to increase patient safety through better continuity of care, better quality of care, more patient-centered care and equal care [1–3]. While digital nursing documentation can be structured in different ways, the majority of electronic nursing records has been shown to be organised according to the nursing process [2, 4]. The nursing process as a problem-solving approach has been described as an important tool for nursing practice, increasing critical thinking and reasoning for care [5]. The step-wise process includes assessment of the patient, nursing diagnoses and goals, planning, nursing interventions and evaluation of practice. This forms the basis of nursing documentation as a care plan [6]. In nursing practice the patient may have several problems, with each requiring different descriptions of nursing problems, or diagnoses [5].

A systematic review of systematic reviews [7] showed that most of the included reviews indicated higher quality in nursing documentation when aligned with the nursing process. To be considered as high quality, the documentation should demonstrate a logical relationship between the steps of the nursing process, and all elements should be clearly stated [2, 8, 9]. However, nursing documentation often do not adhere to these criteria [2, 8–10] and this lack of quality in documentation may lead to poorer care [1].

In research, an audit instrument is commonly used to evaluate the quality of nursing records [2, 9, 10]. Several audit instruments have been developed, measuring some or all steps of the nursing process [11]. Measuring nursing documentation using an audit instrument in combination with interventions may improve nursing documentation [12]. The aim of this project was therefore to assess the quality of electronic nursing records in a Norwegian residential care home using a standardized audit tool, and if necessary, implement a tailored change strategy to improve documentation practice.

## Methods

We performed a criteria-based clinical audit as described by the Health Care Quality Improvement Partnership [13]. The project process consisted of setting criteria and standards according to research and legal demands, applying a suitable audit tool, measuring baseline documentation quality, implementing tailored interventions based on identified barriers and facilitators, and re-measurement of documentation quality.

### Setting

The project took place at two wards in a Norwegian community care centre, which was organised as a residential care home for people with dementia needing 24-hour nursing care. The regular staff included eight nurses (19 %), 14 assistant nurses (35 %), and 20 assistants without a health care degree (47 %). The staff organization was based on a primary care model [14].

The community care centre applied an electronic patient records system that integrated the nursing process in care planning, with freewriting for diagnoses, aims, interventions, evaluation and daily reports. According to local guidelines, nurses and assistant nurses were responsible for the development of nursing care plans, while all staff compiled progress reports in the electronic patient records.

### Criteria, standard and audit tool

Criteria and standard were defined in line with the step-wise nursing process and the nursing care plan [6], the N-Catch II audit instrument [15] and Norwegian regulations for patient records [16].

The N-Catch II was culturally adapted and translated into Norwegian [17] from the valid and reliable Dutch D-Catch instrument [11]. It is designed to assess electronic written nursing documentation and includes a quantitative (e.g presence of relevant information), and a qualitative (e.g. correctness of language) assessment of documentation. In this audit, we focused on the quantitative assessment of the documentation, assessing the following steps (criteria) in the nursing process; ”Nursing assessment on admission”, “Nursing diagnoses”, “Aims for nursing care”, “Nursing interventions”, and “Evaluation/progress report” (Table 1). Each step was scored on a scale from 0 to 3, with zero indicating inadequate or missing documentation and three complete documentation.

**Table 1:** Criteria and standards for nursing documentation in electronic patient records

The nursing process	Criteria (N-Catch)	Evidence base	Standard
Nursing assessment on admission	The patient’s health history, the reason for admission and the patient’s health status should be completely documented	Wang, Hailey & Yu [9], Norwegian Ministry of Health and Care Services [16]	100 % of admission notes should fulfill these criteria (N-catch score = 3)
Nursing diagnoses	Nursing problem, etiology and symptoms should be clearly described.	Wang, Hailey & Yu [9], Müller-Staub et al. [8], Norwegian Ministry of Health and Care Services [16]	100 % of nursing care plans should fulfill these criteria (N-catch score = 3)
Aims for nursing care	Aims should relate to nursing diagnosis, be measurable, realistic and describe a desired situation for the patient in the future.	Wang, Hailey & Yu [9], Norwegian Ministry of Health and Care Services [16]	100 % of nursing care plans should fulfill these criteria (N-catch score = 3)
Nursing interventions	Nursing interventions should be specific and relate to nursing diagnosis and aims.	Wang, Hailey & Yu [9], Norwegian Ministry of Health and Care Services [16]	100 % of nursing care plans should fulfill these criteria (N-catch score = 3)
Evaluation/ progress reports	Evaluations/ progress reports should assess the patients’ health status and relate to nursing diagnoses, aims and nursing interventions.	Jefferies, Johnson &Griffiths [18], Norwegian Ministry of Health and Care Services [16]	100 % of evaluation reports should fulfill these criteria (N-catch score = 3)

To assess inter-rater reliability for the audit instrument, the first author (PM) and a nurse individually scored all criteria in seven patient records. In advance, the raters met for 45 min to reach a mutual understanding of the instrument. Agreement was calculated as the proportion of equal responses on all parts defining a criteria [19], and ranged from 0 % on the criterion “Nursing assessment on admission” to 87 % on “Aims for nursing care” (Table 2). The reasons for discrepancies between raters were different interpretation of items in the N-Catch II and dissimilar interpretation of information in the nursing records. The raters reached consensus based on a discussion of scores and clarification of audit tool.

**Table 2:** Inter-rater reliability of the audit instrument based on seven patient records

Item of audit instrument	Agreement (%)
Nursing assessment on admission	0
Nursing diagnoses	54
Aims for nursing care	87
Nursing interventions	59
Evaluation/progress reports	56

### Data collection

A power analysis informed that at least 33 patient records were needed to estimate a mean with 95 % confidence and precision equal to 0.2, this when assuming an expected population standard deviation (SD) of 1.5 and a population size of 38. For comparison of mean values at audit and re-audit, a total sample size of 72 (36 in each group) was needed to detect a difference in means of 1 unit and assuming SD equal to 1.5. This to achieve a power of 80 % and a two-sided significance level of 5 %. The software R [20] as applied for sample size calculations using the sample.size.means function in the Samplingbook package [21], and the pwr.t.test function in the pwr package [22].

We included nursing records for residents at the two included wards. If nursing care plans had been initiated before the study period, the records were included if the patients` health problem was still present. We excluded assessments, transfer notes, archived or former edits of nursing care plans, and daily reports written by others than the regular staff at the two wards.

Baseline data was collected retrospectively from electronic patient records during January to March 2018. In this period, 39 patient records were eligible and all, but one fulfilled the inclusion criteria. The re-audit was based on 38 patient records during March to June 2019.

### Data analyses

Frequency bar charts were established to compare the distribution of score values at audit and re-audit. We presented mean scores at audit and re-audit, and mean score differences (MD) with 95 % confidence intervals (CI) for each criteria. Changes in mean scores were tested with the independent sample t-test. P-values less than 0.05 indicated statistical significance. The statistical software IBM SPSS Statistics version 25 [23] was used for the statistical analyses.

### Implementation strategy

Based on findings from a literature search for implementation research, audit findings and identified barriers, we designed a multifaceted implementation strategy [24], with interventions tailored to feedback [25], printed educational materials [26] and local opinion leaders [27]. The first author (PM) worked as a nurse at the facility and performed the clinical audit as a masters’ degree. He led the project and the implementation process in collaboration with leaders at the community care centre.

The first step of our strategy was feedback on baseline results to staff and leaders in small groups. We held one session with all leaders and five staff sessions where 47 % attended. The staff sessions were held at lunch time and in-between shift changes, and the staff was encouraged by the head nurse to participate. At the end of all sessions, we performed a brainstorming where we asked the staff to elaborate barriers and facilitators for changing documentation practice. Further, we performed a root-cause analysis and described interventions tailored to local barriers in collaboration with leaders at the community centre.

The identified local barriers were related to lack of knowledge and skills, lack of resources (computers) and insufficient time for writing patient records (Table 3). Local opinion leaders were identified as head nurses, assistant head nurses and experts in electronic nursing documentation software. To tailor the training in electronic documentation, we applied checklists to identify the nurses’ and assistant nurses’ knowledge and skills in the applied software and electronic patient records documentation. Further, we developed educational material and cards with documentation guidelines to be available as reminders by computers, supplied the wards with additional computers, and encouraged staff to organize time for documentation. At the same time, the leaders at the community care centre performed an educational session where staff were introduced to updated terminology and criteria for nursing documentation, in line with a national quality project in nursing documentation [28].

The implementation period lasted from October 2018 to March 2019. We started by updating opinion leaders and providing feedback sessions to staff. In December, the leaders of the care centre held mandatory educational sessions to all staff, and by February the workstations were upgraded. The other interventions were executed concurrently during the implementation period.

**Table 3:** Identified barriers and tailored implementation interventions

Identified barriers	Interventions	Local strategy
Lack of knowledge and skills	Feedback of audit findings [25] Information and education, individually and in groups [26] Local opinion leaders [27]	Perform feedback of audit findings at lunch and in-between shift meetings. Perform educational sessions with information of updated criteria and terminology [28]. Use local opinion leaders to motivate and teach documentation skills. Apply checklist to identify nurses’ and assistant nurses’ knowledge and skills in applied software and electronic patient records documentation. Develop educational material and cards with documentation guidelines to be available as reminders by computers.
Lack of available resources (computers)	Provide satisfying workstations for documentation	Supply each ward with additional computers. Establish workstations (computers side by side) where staff may collaborate on documentation
Insufficient time for writing patient records	Organize time for documentation	Organize time and stress the importance of documentation. Motivate staff to engage in written documentation practice.

### Ethical considerations

The need for ethics approval and consent to participation was deemed unnecesserary according to national regulations [29]. We received approval from the local Data Protection Officer acting for the Norwegian Data Protection Authority.

## Results

**Fig. 1:**
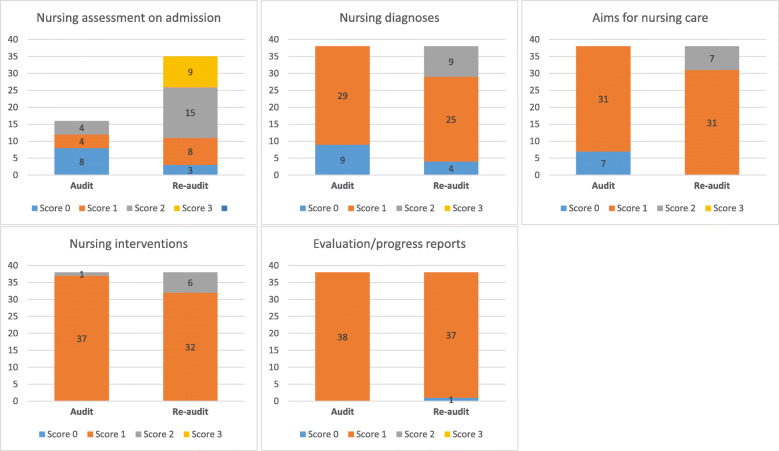
Distribution of scores by criteria to measure adherence to recommended documentation practice in a residential care home. Analyses were based on 38 patient records at audit and re-audit, respectively, except for “Nursing assessment on admission” where 16 records were analyzed at audit and 35 records at re-audit

In all, 38 patient records were assessed at audit and at re-audit. At audit, standard (score 3) was not met for any of the criteria (Fig. 1) and score 2 was obtained for a few records only. This entailed one record for the criterion “Nursing interventions” and four records for the criterion “Nursing assessment on admission”. At re-audit, standard was reached for one criterion only, namely for “Nursing assessment on admission” where 9 records had a score equal to 3 (Fig. 1). Still, we observed an improvement in adherence to recommended documentation practice over time. A comparison of mean scores at audit and re-audit showed a statistically significant improvement (*p* < 0.001) for all criteria except “Evaluation/progress report” (*p* = 0.6) (Table 4). The most evident positive change was for “Nursing assessment on admission” where scores on average increased with 1.1 units (95 % CI 0.56–1.65). Also, while the proportion of missing assessment notes was 58 % at audit it had decreased to 8 % at re-audit.

**Table 4:** Adherence to recommended documentation practice in 38 patient records at a residential care centre

Criteria	Audit	Re-audit	Change	
	Mean (95 % CI)	Mean (95 % CI)	MD ^a^ (95 % CI)	*p*-value
Nursing assessment on admission ^b^	0.8 (0.3–1.2)	1.9 (1.5–2.2)	1.1 (0.6–1.7)	< 0.001
Nursing diagnoses	1.1 (0.9–1.3)	1.7 (1.5–1.8)	0.5 (0.3–0.7)	< 0.001
Aims for nursing care	0.4 (0.3–0.6)	1.0 (0.8–1.1)	0.5 (0.3–0.8)	< 0.001
Nursing interventions	0.9 (0.8–1.1)	1.4 (1.3–1.6)	0.4 (0.2–0.6)	< 0.001
Evaluation/progress reports	0.8 (0.6–1.0)	0.9 (0.8–1.0)	0.0 (-0.2–0.1)	0.6
^a^*MD* Mean difference				
^b^ “Nursing assessment on admission“ was missing in 22 records at audit and 3 records at re-audit. The N-Catch II did not provide a scoring option for missing values for these cases				

## Discussion

This quality improvement project identified a knowledge gap in electronic nursing documentation at a residential care home. None of the audited patient records fulfilled standards for recommended documentation practice. However, an improvement was noted after implementation of a tailored multifaceted intervention strategy for all steps of the nursing process, except for “Evaluation/progress report”.

Our findings are in line with other recent investigations with settings in nursing homes and residential care centres [30] reflecting that an earlier call for research into this area [9] is still relevant. The barriers identified in our study, lack of knowledge and skills, and lack of available resources in terms of time and hardware, coincided with those reported in other studies on implementation of electronic health records in long-term care facilities [1, 31]. We observed that lack of knowledge and skills did not refer only to knowledge of what constituted quality nursing documentation, but also to navigation of the software system.

As has been reported in other studies [2], our electronic patient record system was not originally designed for nursing documentation [32].

In contrast to Nøst and colleagues [12], we did not observe improved documentation practice for the step “Evaluation/progress report”. This may in part be due to the software structure. In the electronic records, nursing diagnoses, aims and nursing interventions were not accessible when writing evaluation and progress reports. Thus, the staff could not easily read the nursing care plan when they were writing the reports. Lack of knowledge in nursing documentation may also be an issue. While all staff, including assistants without a health care degree, wrote evaluation and progress reports, only nurses and assistant nurses developed the nursing care plans. In this project, we did not specifically measure the internal relationship between nursing care plans and evaluation and progress reports. Such an investigation could, however, have clarified the results, and possibly pointed to interventions that could lead to a more complete implementation of electronic nursing records.

It has been argued that a standardized nursing language might provide better continuity of care and thus contribute to patient safety, but heterogeneity among studies prevented a reliable effect estimate [2]. A systematic review of systematic reviews (umbrella review) indicated that high-quality nursing documentation depended on documentation being aligned with the nursing process, use of standard terminologies and user-friendly formats [7]. A recent survey among nurses also suggested an association between variations in use of standardized terminologies and poor user-friendliness, with seeing electronic health records as less beneficial [33]. An integrated review investigating electronic patient records as facilitator of care in nursing homes, stated a need for a focus on the type of documentation system used, and further for research among end users to identify the optimal system characteristics for use in this type of setting [34]. A scoping review [35] also pointed to a general lack of research into patient safety and patient safety culture in nursing homes, and in residential care homes in particular.

### Limitations

It is a strength of this project that interventions were tailored to identified barriers. An umbrella review [36] deemed the certainty of evidence as moderate regarding tailored interventions leading to a probable increase in adherence to clinical practice guidelines. The same conclusion was made for the specific interventions applied in this study, namely audit and feedback, use of local opinion leaders and educational meetings [35]. Audit and feedback have further been shown to be more effective in cases with a low baseline performance [25], as was the case in this study.

There were some limitations in this study. Firstly, the study was limited to an evaluation of quantitative aspects of nursing documentation only. As with D-Catch [11], the N-Catch II also define quality aspects of documentation. Incorporating a qualitative evaluation could clearly have added important information on documentation practice. Secondly, the scoring system of N-Catch II was experienced as unclear and may explain the initial poor inter-rater agreement. Previous studies have stated a need for adjustment of both design and text of the N-Catch II [12], and possibly a longer training period than 45 min should have been allotted in this study. However, due to a busy working schedule this was not feasible. Investigations of the original D-Catch instrument showed high inter-rater agreement, but this was following a 20-hour training period [11]. Similarly, with the N-Catch instrument Instefjord and colleagues [37] and Johnsen and colleagues [38] indicated a more comprehensive understanding of the instrument among the reviewers. Thirdly, while the N-Catch has been tested for reliability [38], other psychometric measurements have so far not been reported for this instrument, and the updated N-Catch II instrument has not yet been tested for psychometric measurements. Finally, we experienced low staff attendance at sessions where audit findings were presented. Although there was some verbal dissemination of audit findings to non-attending staff, providing written feedback could have increased awareness. Also, the total implementation strategy was designed to engage all staff. Still, the uptake of interventions is unclear.

## Conclusions

The quality of nursing documentation was not satisfactory, but the study indicated that a criteria-based clinical audit could improve the quality of nursing documentation if combined with multifaceted tailored interventions that address determinants of practice. Further research is needed into what constitutes quality nursing documentation and how to best measure nursing documentation.

## Data Availability

The datasets used and analyzed during the current study are not publicly available due to confidentiality in patient´s medical records but are available from the corresponding author on reasonable request.

## Abbreviations

SD: Standard Deviation
MD: Mean Difference
CI: Confidence interval

## References

[1] Meißner A, Schnepp W. Staff expericenes within the implementation of computer-based nursing records in residential aged care facilities: a systematic review and synthesis of qualitative research. BMC Med Informatics Decision Making. 2014; 14(54). 10.1186/1472-6947-14-54. 10.1186/1472-6947-14-54 PMC4114165 24947420

[2] Saranto K, Kinnunen UM, Kivekäs E, Lappalainen AM, Liljamo P, Rajalahti E (2014). Impacts of structuring nursing records: a systematic review. Scand J Caring Sci.

[3] Urquhart C, Currell R, Grant MJ, Hardiker NR. Nursing record systems: effects on nursing practice and healthcare outcomes. Cochrane Database Syst Reviews. 2009; (1). Available from: 10.1002/14651858.CD002099.pub2. 10.1002/14651858.CD002099.pub2 19160206

[4] Häyrinen K, Saranto K, Nykänen P (2008). Definition, structure, content, use and impacts of electronic health records: a review of the research literature. International journal of medical informatics.

[5] Blair W, Smith B (2014). Nursing documentation: Frameworks and barriers. Contemp Nurse.

[6] Björvell C, Thorell-Ekstrand I, Wredling R (2000). Development of an audit instrument for nursing care plans in the patient record. Q Health Care.

[7] De Groot K, Triemstra M, Paans W, Francke A. Quality, criteria, instruments, and requirements for nursing documentation: A systematic review of systematic reviews. J Adv Nurs [Internet]. 2019; 75:[1379-93 pp.]. 10.1111/jan.13919 30507044

[8] Müller-Staub M, Lavin MA, Needham I, Van Achterberg T (2006). Nursing diagnoses, interventions and outcomes – application and impact on nursing practice: systematic review. J Adv Nurs.

[9] Wang N, Hailey D, Yu P (2011). Quality of nursing documentation and approaches to its evaluation: a mixed-method systematic review. J Adv Nurs.

[10] Saranto K, Kinnunen U-M. Evaluating nursing documentation – research designs and methods: systematic review. J Adv Nurs. 2009; 65(3):464 – 76. Available from: 10.1111/j.1365-2648.2008.04914.x. 10.1111/j.1365-2648.2008.04914.x 19222644

[11] Paans W, Sermeus W, Nieweg RMB, van der Schans CP (2010). D-Catch instrument: development and psychometric testing of a measurement instrument for nursing documentation in hospitals. J Adv Nurs.

[12] Nøst TH, Frigstad SA, André B (2017). Impact of an education intervention on nursing diagnoses in free-text format in electronic health records: A pretest-posttest study in a medical department at a university hospital. Nordic J Nurs Res.

[13] Burgess R, editor. New principles of best practice in clinical audit. 2nd ed. ed. Oxford: Radcliffe Publishing; 2011.

[14] Edberg A-K, Ehrenberg A, Friberg F, Wallin L, Wijk H, Öhlèn J (2010). Omvårdnadens grunder - En specialutgåva för sjuksköterskor.

[15] Nøst TH, Haugan B, Oppheim AE, Tettum BI, Woldstad K, Mikkelsen J, et al. N-Catch II. Granskningsinstrument for vurdering. av sykepleiedokumentasjon i elektronisk pasientjournal (EPJ). 2013

[16] Regulations on Patient Records [Forskrift om pasientjournal], FOR-2019-03-01-168. (2019).

[17] Nøst TH, Tettum BI, Frigstad SA, Woldstad K, Haugan B, Oppheim AE, et al. D-Catch blir norsk. Sykepleien [Internet]. 2015; 2015(05). Available from: http:/doi.10.4220/Sykepleiens.2015.54056.

[18] Jefferies D, Johnson M, Griffiths R. A meta-study of the essentials of quality nursing documentation. Int J Nurs Pract. 2010; 16(2):112 – 24 pp. 10.1111/j.1440-172X.2009.01815.x 20487056

[19] Polit DF, Beck CT. Nursing Research: generating and assessing evidence for nursing practice. 10th ed. ed. Philadelphia: Wolters Kluwer; 2016.

[20] R Core Team. R: A language and environment for statistical computing.2018. Available from: https://www.r-project.org.

[21] Documentation Samplingbook R. v.1.2.2 [Available from: https://www.rdocumentation.org/packages/samplingbook/versions/1.2.2/topics/sample.size.mean.

[22] Rdocumentation pwr t. test [Available from: https://www.rdocumentation.org/packages/pwr/versions/1.3-0/topics/pwr.t.test.

[23] IBM Corp. IBM SPSS Statistics for Windows. 25.0 ed. Armonk: IBM Corp.; 2017.

[24] Baker R, Camosso-Stefinovic J, Gillies C, Shaw EJ, Cheater F, Flottorp S, et al. Tailored interventions to address determinants of practice. Cochrane Database Syst Reviews. 2015(4). 10.1002/14651858.CD005470.pub3. 10.1002/14651858.CD005470.pub3 PMC7271646 25923419

[25] Ivers N, Jamtvedt G, Flottorp S, Young JM, Odgaard-Jensen J, French SD, et al. Audit and feedback: effects on professional practice and healthcare outcomes. Cochrane Database Syst Reviews. 2012(6). 10.1002/14651858.CD000259.pub3. 10.1002/14651858.CD000259.pub3 PMC11338587 22696318

[26] Giguère A, Légaré F, Grimshaw J, Turcotte S, Fiander M, Grudniewicz A, et al. Printed educational materials: effects on professional practice and healthcare outcomes. Cochrane Database Syst Reviews. 2012(10). 10.1002/14651858.CD004398.pub3 PMC7197046 23076904

[27] Flodgren G, Parmelli E, Doumit G, Gattellari M, O’Brien MA, Grimshaw J, et al. Local opinion leaders: effects on professional practice and health care outcomes. Cochrane Database Syst Reviews. 2011(8). 10.1002/14651858.CD000125.pub4 PMC4172331 21833939

[28] Remlo L, Olsen KJ. Dokumentasjon av helsehjelp - et kompetanse- og kvalitetsforbedringsprogram for kommunene i Troms2018 18 desember 2018. Available from: http://www.utviklingssenter.no.

[29] Lov 2. juli 1999 nr. 64 om helsepersonell m.v., (1999). Available from: https://www.lovdata.no.

[30] Tuinman A, de Greef MHG, Krijnen WP, Paans W, Roodbol PF (2017). Accuracy of documentation in the nursing care plan in long-term institutional care. Geriatr Nurs.

[31] Kruse CS, Mileski M, Alaytsev V, Carol E, Williams A (2015). Adoption factors associated with electronic health record among long-term care facilities: a systematic review. BMJ Open.

[32] Norwegian Directorate of Health [Helsedirektoratet] (2014). Electronic Patient Records in Care Services - Status, challenges and needs [Elektronisk pasientjournal i omsorgstjenesten - status, utfordringer og behov].

[33] De Groot K, De Veer AJE, Paans W, Francke AL (2020). Use of electronic health records and standardized terminologies: A nationwide survey of nursing staff experiences. Int J Nurs Stud.

[34] Shiells K, Holmerova I, Steffl M, Stepankova O (2018). Electronic patient records as a tool to facilitate care provision in nursing homes: an integrative review. Inform Health Soc Care.

[35] Gartshore E, Waring J, Timmons S (2017). Patient safety culture in care homes for older people: a scoping review. BMC Health Serv Res.

[36] Fretheim A, Flottorp S, Oxman AD. Effect of interventions for implementing clinical practice guidelines [Effekt av tiltak for implementering av kliniske retningslinjer]. Oslo: Nasjonalt kunnskapssenter for helsetjenesten; 2015. Available from: https://www.fhi.no/publ/2015/effekt-av-tiltak-for-implementering-av-kliniske-retningslinjer/. 28510383

[37] Instefjord MH, Aasekjær K, Espehaug B, Graverholt B. Assessment of quality in psychiatric nursing documentation – a clinical audit. 2014. 10.1186/1472-6955-13-32 PMC4207848 25349532

[38] Johnsen KF, Ehrenberg A, Fossum M (2014). Dokumentasjon av sykepleie i sykehjem. Nordic Journal of Nursing Research.

